# Retroperitoneal schwannoma during pregnancy: A case report and practice-based overview

**DOI:** 10.1016/j.crwh.2024.e00590

**Published:** 2024-02-28

**Authors:** Stefano Fruci, Anna Fagotti, Silvia Salvi, Pierpaolo Mattogno, Quintino Giorgio D'Alessandris, Federica Totaro Aprile, Antonia Carla Testa, Antonio Lanzone, Liverana Lauretti

**Affiliations:** a UOC di Ostetricia e Patologia Ostetrica, Dipartimento di Scienze della Salute della Donna, del Bambino e di Sanità Pubblica, Fondazione Policlinico Universitario A. Gemelli, IRCCS, L.go Agostino Gemelli 8, Rome 00168, Italy; b Dipartimento Universitario di Scienze della Vita e Sanità pubblica - Sezione di Ginecologia ed Ostetricia - Università Cattolica del Sacro Cuore, L.go Francesco Vito 1, Rome 00168, Italy; c UOC Ginecologia Oncologica, Dipartimento di Scienze della Salute della Donna, del Bambino e di Sanità Pubblica, Fondazione Policlinico Universitario A. Gemelli IRCCS, L.go Agostino Gemelli 8, Rome 00168, Italy; d Department of Neurosurgery, Fondazione Policlinico Universitario A. Gemelli IRCCS, L.go Agostino Gemelli 8, Rome 00168, Italy; e Dipartimento Universitario di Neuroscienze - Università Cattolica del Sacro Cuore, L.go Francesco Vito 1, Rome 00168, Italy

**Keywords:** Schwannoma, Pregnancy, Ultrasound, Magnetic resonance imaging

## Abstract

The retroperitoneum is the rarest site for Schwannomas, tumors that originate from Schwann cells and usually present as benign, slowly growing masses. During pregnancy, the routine application of ultrasound for fetal assessment has led to an increased rate of detection of maternal asymptomatic masses, notably including the retroperitoneal ones. While most of these masses prove to be benign, it is imperative to consider the potential for malignancy. This report presents a rare case involving a woman diagnosed with bilateral adnexal cysts and a pre-sacral retroperitoneal mass during the first trimester of pregnancy. Surgical intervention was employed to remove ovarian tumors, and a biopsy was performed on the non-adnexal tumor to determine its nature. The histological examination revealed a bilateral borderline seromucinous tumor in the ovaries and identified a Schwannoma in the sacral mass. Despite the considerable size of the pre-sacral mass, which significantly impacted the patient's quality of life, successful measures were taken to achieve a near-term pregnancy, culminating in the delivery of a healthy baby. Subsequently the patient underwent neurosurgical treatment of the substantial pre-sacral Schwannoma. The discovery of a Schwannoma during pregnancy can evoke concerns among healthcare practitioners, touching upon potential malignancy risks, accelerated tumor growth, and impacts on fetal well-being. This paper provides a comprehensive, practice-based overview of these critical aspects.

## Highlights

• Ultrasound may be of benefit in diagnosing retroperineal masses during pregnancy.
• The growth of benign tumors such as Schwannomas does not accelerate during pregnancy.
• Voluminous pelvic masses do not hamper childbearing.

## 1 Introduction

Schwannomas, also called neurinomas, are tumors that affect cranial and spinal nerves, originating from Schwann cells. Characterized by relatively slow growth, neurinomas are usually benign: in less than 1% of cases do they become malignant. They usually appear as sporadic isolated tumors, whereas multiple tumors may occur in genetic syndromes like neurofibromatosis type 2 (NF2) and Schwannomatosis. Schwannomas can occur in many locations, but the more common sites are head, neck, and flexor surfaces of the extremities. Retroperitoneal Schwannomas are extremely rare, accounting for only 0.5% to 5% of all cases [1]. They can reach considerable dimensions because symptoms develop late and are not specific; moreover, their location makes diagnosis more difficult.

During pregnancy, the enhanced use of prenatal ultrasound for fetal assessment has increased the rate of detection of maternal asymptomatic masses, including retroperitoneal ones. Although most masses are benign, the possibility of a malignant tumor must always be considered. This report describes the rare case of a woman with bilateral adnexal cysts and a retroperitoneal pre-sacral mass diagnosed during the first trimester of pregnancy. Managing multiple pelvic masses during pregnancy poses a complex scenario; some clinical challenges and ethical dilemmas are here examined, and a practice-based overview on this topic is also presented.

## 2 Case Presentation

A 35-year-old pregnant woman at the 11th gestational week was evaluated for recent findings of bilateral ovarian masses and a pre-sacral retroperitoneal mass. During the previous two years, she had complained of episodes of severe pain in the legs, which had always been treated with non-steroidal anti-inflammatory drugs. For the same reason, she was hospitalized at the 9th gestational week, when a transvaginal pelvic ultrasound (TV-US) and pelvic magnetic resonance imaging (MRI) were performed. The scans documented the presence of the ovarian masses and showed, on the left posterior side of the pelvis, an expansive mass of nearly 10 cm, originating from the spinal roots, S1-S2. Tumor markers were measured to assess whether the adnexal masses were secondary in nature; these showed a raised Ca 19.9 level, while Ca 125 and Ca 15.3 were both in the normal range.

The patient was hospitalized for further medical investigations and obstetric ultrasound, TV-US and non-contrast MRI were performed. The TV-US showed a solid multilocular mass of 69x49x61 mm in the right adnexal region, with “low level” cyst content and an irregular internal surface due to the presence of numerous papillae, the largest measuring 14x17x14 mm, moderately vascularized at color Doppler, with residual ovarian parenchyma measuring 26x10x20 mm; in the left adnexal region, a solid multilocular mass formation of 107x54x72 mm was also detected, with “low level” cyst content and irregular internal surface due to the presence of a moderately vascularized papilla of 14x25x20 mm. The risk of malignancy of the target lesion was estimated to be 45% according to the International Ovarian Tumor Analysis Adnex Model. The ADNEX (Assessment of Different NEoplasias in the adneXa) model [2] is a risk model based on multinomial logistic regression; the predictor variables used are the woman's age (years), the maximum diameter of the lesion (mm), the maximum diameter of the largest solid component (mm), the number of papillary projections (ordinal), the presence of acoustic shadows, the presence of ascites, the presence of more than 10 cyst locules, the type of medical center (oncology center vs other) where the investigation has been performed and the serum CA 125 values (IU/L).

**Fig. 1:**
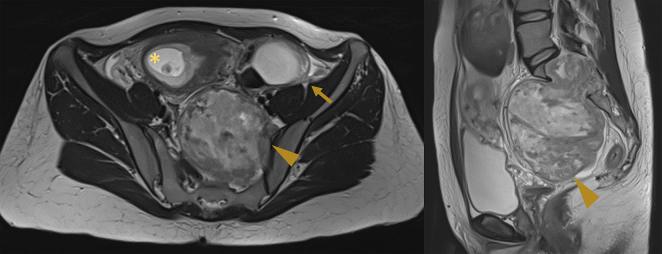
No-contrast magnetic resonance imaging during pregnancy. On the left, an axial scan showing the viable fetus (*), the multilocular solid left ovarian mass (arrows) and the retroperitoneal Schwannoma (arrowhead); on the right, a sagittal scan with the retroperitoneal Schwannoma (arrowhead).

Moreover, posterior to the cervix, a solid mass of 97x71x99 mm was detected, which appeared to be fixed to and not separable from the sacrum and was characterized by regular margins, and an inhomogeneous and poorly vascularized echostructure. The non-contrast MRI (Fig. 1) showed: the viable fetus (*), the multilocular solid left ovarian mass (arrows) and the voluminous (12 × 10 cm) solid tumor (arrowhead) deforming the uterus and extending into the spinal canal at the sacral level.

To understand the nature of the ovarian lesions and to reduce the steric hindrance caused by the two ovarian neoformations, it was decided at a multidisciplinary case review, with approval by the ethics committee, to opt for surgical removal during pregnancy with simultaneous biopsy of the tumor originating from the nerve sheath. At 12 weeks of gestation, the patient underwent a left salpingo-oophorectomy, right unilateral cystectomy, omental biopsy, and a biopsy of the pre-sacral neoformation, with a laparoscopic/laparotomic approach (initial laparoscopy later converted to laparotomy). The primary aim of the exploratory laparoscopy was to rule out peritoneal carcinomatosis, which is not always easily detectable through diagnostic imaging, especially as pregnancy is a counter-indication to the use of contrast-enhanced computed tomography. Upon confirming the absence of peritoneal carcinomatosis and recognizing the substantial size of the adnexal and pre-sacral masses, the decision was made to transition to a laparotomy for the comprehensive surgical intervention. The histological examination uncovered a Schwannoma in the pre-sacral mass; the right ovarian neoformation was found to be a borderline sero-mucinous ovarian tumor, and the left adnexal mass was a borderline sero-mucinous tumor in the context of an endometriotic cyst. The omental tissues were free from atypia.

**Fig. 2:**
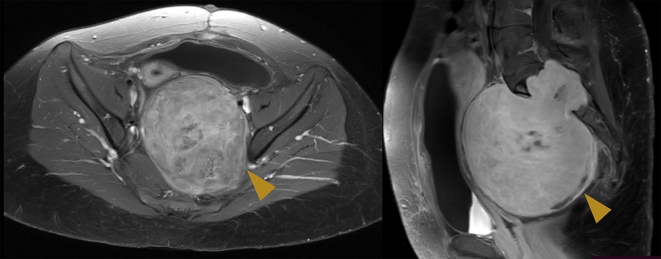
Magnetic resonance imaging after pregnancy. On the left, an axial scan showing the same retroperitoneal Schwannoma (arrowhead); on the right, a sagittal scan with the retroperitoneal Schwannoma (arrowhead).

Subsequently, the pregnancy was uneventful, except the worsening maternal pain prompted a planned a C-section at the 37th gestational week. The C-section was chosen since the pre-sacral Schwannoma prevented the normal descent of the fetal head; the patient was discharged on the third day without complications and the histological examination of the placenta was negative for atypical and/or cancerous cells. MRI performed after delivery did not reveal any changes in the Schwannoma's volume (Fig. 2). Seven months later, neurosurgical removal was successfully performed. Surgery lasted 8 h, intraoperative blood loss was 3 l and the patient was transfused with 4 units of concentrated red blood cells and 3 units of plasma. The definitive histological examination of the pre-sacral lesion confirmed the diagnosis of Schwannoma (grade I according to World Health Organization 2016).

## 3 Discussion

There are few reports of pelvic Schwannomas diagnosed during pregnancy. While the routine use of ultrasound scans has increased the rate of detection of retroperitoneal masses during pregnancy, diagnosis is still challenging [3]. Differential diagnoses include chordoma, dermoid tumor, chondrosarcoma, sarcoma, and lymphoproliferative tumor. With ultrasound, the fluid/multiloculated content or the relationship with major nerve trunks might hint at a Schwannoma; however, the relationship with minor pelvic nerves or with the lumbosacral nerve roots are better recognized with MRI, which also visualizes the enlargement of radicular foramina and the intrathecal tumor extension if present. Then, lumbo-sacral MRI (without contrast is adequate) should be performed to confirm or rule out a Schwannoma in the case of a pelvic, not adnexal, retroperineal mass, during the gestational period. In the case reported here, a biopsy was performed.

Healthcare practitioners after finding a Schwannoma during pregnancy will be concerned about: 1) the risk of malignant degeneration; 2) an acceleration in tumor growth due to the gestation; and 3) fetal health and growth risks. Concerning the risk of malignancy, there are no reports of benign pelvic Schwannomas degenerating to malignant peripheral nerve sheath tumor (MPNST) during pregnancy. The only case of MPNST during pregnancy concerned a voluminous intrathoracic tumor that rapidly developed and evolved in a patient affected by neurofibromatosis type 1 (NF1) [4]: the authors reported that surgical tumor removal was needed during pregnancy because of the persistent and intractable pain coupled with the clinical conditions worsening. In the present case, however, there was no evidence that the tumor had started as benign and had changed to a malignant form during pregnancy. Regarding the risk of an acceleration in tumor growth during pregnancy, for pelvic locations very few cases are available in the literature [[5], [6], [7]]. In one case the patient chose to start a second pregnancy before the tumor was removed, and no changes in tumor volume were detected after the conclusion of the two pregnancies [5]. However, enlargement of Schwannomas during pregnancy has been reported for an orbital location [6] and for a vestibular nerve origin [7]: in the latter, a rapid worsening of neurological symptoms caused by the huge dimensions of the tumors was described in a patient who presented during the second trimester of pregnancy; the size required its surgical removal during pregnancy, and may be better explained by fluid retention and the metabolic changes of the gestational period more than by the only acceleration of tumor growth.

Concerning potential risks to the fetus, particularly for pelvic sites, a low likelihood of adverse outcomes is suggested by the present and similar cases reported in the literature where pregnancy was uneventful. An elective C-section is usually requested since pelvic masses should be considered a potential obstruction in vaginal delivery.

This report helps to clarify the relevance of ultrasound in diagnosing retroperineal masses during pregnancy and the role of MRI in defining the relationship with vertebral foramina and the nervous structures. If a malignancy is suspected, surgical removal and/or biopsy can be safely undertaken. Nevertheless, pelvic benign tumors do not hamper pregnancy even if of large dimensions: in particular, pelvic Schwannomas are slow-growing benign tumors and do not seem to enlarge during pregnancy.
